# Cannabinoids Facilitate the Swallowing Reflex Elicited by the Superior Laryngeal Nerve Stimulation in Rats

**DOI:** 10.1371/journal.pone.0050703

**Published:** 2012-11-27

**Authors:** Rahman Md. Mostafeezur, Hossain Md. Zakir, Hanako Takatsuji, Yoshiaki Yamada, Kensuke Yamamura, Junichi Kitagawa

**Affiliations:** Division of Oral Physiology, Department of Oral Biological Science, Niigata University Graduate School of Medical and Dental Sciences, Niigata, Japan; CNRS - Université Aix Marseille, France

## Abstract

Cannabinoids have been reported to be involved in affecting various biological functions through binding with cannabinoid receptors type 1 (CB1) and 2 (CB2). The present study was designed to investigate whether swallowing, an essential component of feeding behavior, is modulated after the administration of cannabinoid. The swallowing reflex evoked by the repetitive electrical stimulation of the superior laryngeal nerve in rats was recorded before and after the administration of the cannabinoid receptor agonist, WIN 55-212-2 (WIN), with or without CB1 or CB2 antagonist. The onset latency of the first swallow and the time intervals between swallows were analyzed. The onset latency and the intervals between swallows were shorter after the intravenous administration of WIN, and the strength of effect of WIN was dose-dependent. Although the intravenous administration of CB1 antagonist prior to intravenous administration of WIN blocked the effect of WIN, the administration of CB2 antagonist did not block the effect of WIN. The microinjection of the CB1 receptor antagonist directly into the nucleus tractus solitarius (NTS) prior to intravenous administration of WIN also blocked the effect of WIN. Immunofluorescence histochemistry was conducted to assess the co-localization of CB1 receptor immunoreactivity to glutamic acid decarboxylase 67 (GAD67) or glutamate in the NTS. CB1 receptor was co-localized more with GAD67 than glutamate in the NTS. These findings suggest that cannabinoids facilitate the swallowing reflex via CB1 receptors. Cannabinoids may attenuate the tonic inhibitory effect of GABA (gamma-aminobuteric acid) neurons in the central pattern generator for swallowing.

## Introduction

Cannabinoids (terpenophenolic compounds found in the Cannabis plant, *Cannabis sativa*) have been reported to affect multiple biological functions including appetite, food intake and energy metabolism [1], [2], [3], [4]. Investigations into the biological basis of the multiple effects of cannabinoid have yielded important breakthroughs in recent years. One such fact is that the actions of cannabinoids are mediated by binding with specific receptors namely the cannabinoid receptor 1 (CB1) and cannabinoid receptor 2 (CB2). Various reports have described the critical role of cannabinoids and their endogenous ligands which regulate energy balance and food intake via CB1 receptors of the hypothalamus and limbic structures in the central nervous system (CNS), and through mechanisms involved in adipose tissue and the intestinal system in the periphery [2], [5], [6]. CB1 receptors are found abundantly throughout the CNS including in the brainstem [7], [8], whereas CB2 receptors are found primarily in the immune system [9], [10]. The Brainstem CB1 receptors are mostly located in areas relevant to feeding, such as the nucleus tractus solitarius (NTS) and other nuclei of the dorsal vagal complex (DVC) [11], [12], [13], [14], [15]. Several studies have revealed the functional role of CB1 receptors in the DVC in regulating the gastrointestinal autonomic functions including gastrointestinal vagal reflexes. For example, CB1 receptors regulate the cannabinoid-mediated anti-emetic effects [14], [15], [16], [17] and digestive motor activity [18], [19], and inhibit transient lower esophageal sphincter relaxations [12], [20], [21]. However, the functional role of CB1 receptors in the NTS in this respect is still elusive.

Swallowing is an essential motor component of feeding behavior and is a complex reflex that causes the propulsion of food from the oral cavity into the stomach through the pharynx and esophagus [22]. It is generally well known that swallowing generated by the central pattern generator is located in the NTS [22]. Because of the presence of CB1 receptors in the NTS and the involvement of CB1 receptors in gastrointestinal autonomic functions, we hypothesized that cannabinoids may play crucial role in regulating the swallowing function, and that the action of cannabinoid is mediated by the binding of CB1 receptors in NTS. Based on this assumption we evaluated the effect of the cannabinoid receptor agonist (WIN 55,212-2) on the swallowing reflex elicited by electrical stimulation of the superior laryngeal nerve (SLN), a branch of the vagus nerve [23], [24], [25], [26], [27], in anesthetized rats. In the present study we have demonstrated that cannabinoid facilitate the swallowing reflex elicited by electrical stimulation of the SLN, and that the facilitatory effect of cannabinoid may be mediated by CB1 receptors.

## Methods

### Animal Preparation

A total of 75 male Sprague-Dawley rats weighing 250–300 g were used in the present study. The experiments were carried out in accordance with the “Principles of Laboratory Animal Care” (NIH publication #86-23, revised 1996). The animal protocols were approved by the Intramural Animal Care and Veterinary Science Committee of Niigata University.

The rats were deeply anesthetized with urethane (1.0–1.5 g/kg, administered intraperitoneally). The adequacy of the anesthesia was checked by noxious pressing the hind paw in order to determine if a withdrawal reflex was evoked, and if so, a supplementary dose of urethane was given. After the anesthesia, the rats were fixed in the supine position with adhesive tape and their body temperatures were maintained at 36–37°C with a heating pad. For intravenous infusion of the drugs, the femoral vein was exposed, and then a plastic infusion tube was implanted through the femoral vein. A midline incision was made in the ventral surface of the neck, and the trachea was cannulated to maintain respiration. To record EMG activity, the mylohyoid muscle was exposed and a pair of bipolar electromyographic (EMG) electrodes (Enamel-Nichrome wire) was implanted in the exposed mylohyoid muscle. Swallowing was identified by EMG activity of the mylohyoid muscle and by visual observation of laryngeal movement. Unilateral SLN was exposed by blunt dissection of the sternothyroid muscle and bipolar platinum wire electrodes were fixed onto the central cut end of the SLN. Liquid silicone was poured over the muscle and electrodes to fix the electrode with the nerve. Additionally, silicone prevents the dryness of the nerve, and allowing the nerve to be used for a long term experiment.

### Nerve Stimulation to Evoking Swallowing Reflex and the Intravenous Infusion of Drugs

**Figure 1:**
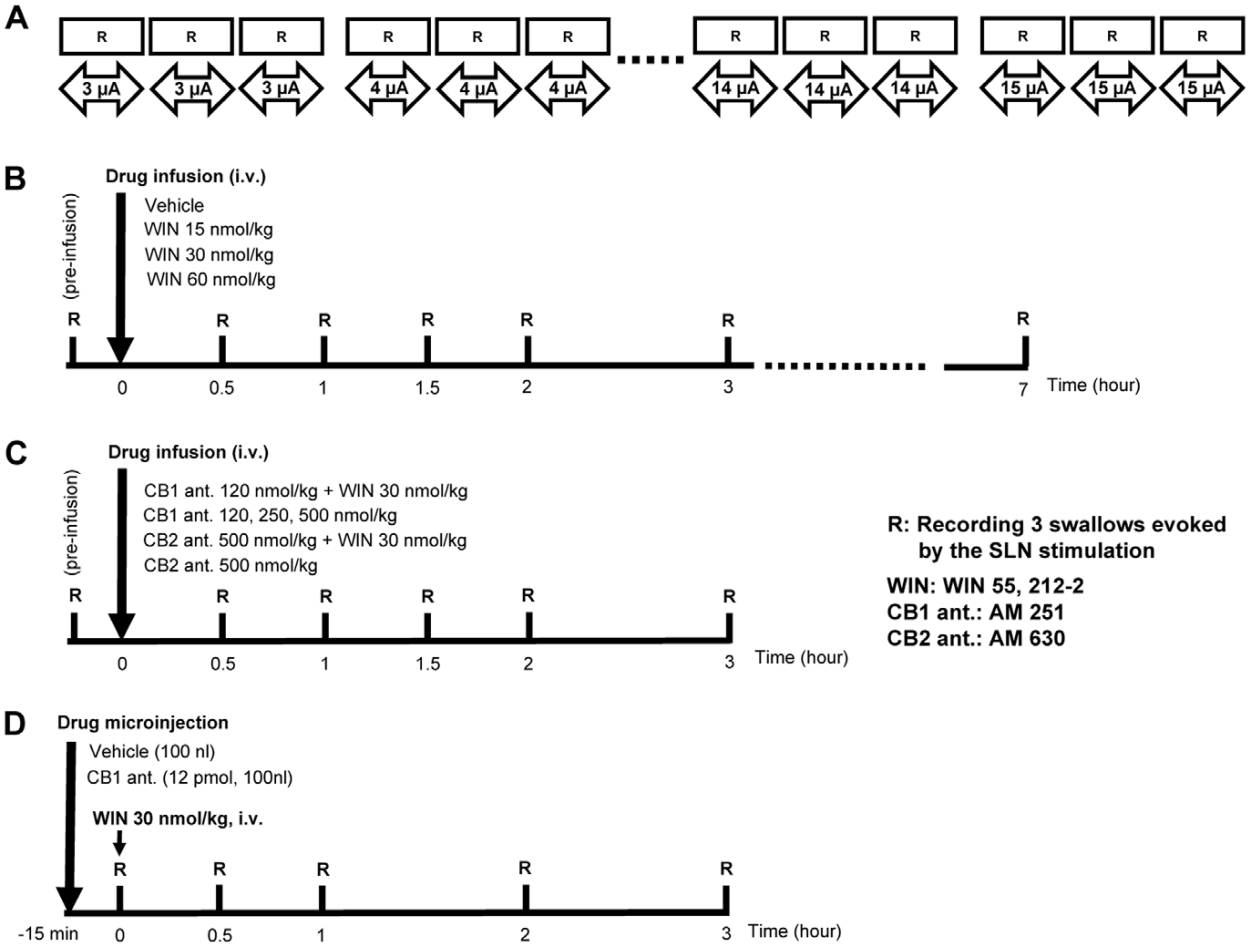
Schematic illustration of the present experimental protocol. The experimental procedure to investigate relationships between the intensity of electrical stimulation and the swallowing reflex (A). The time-course of recording points for the swallowing reflexes before and after intravenous infusion (B and C) of drugs or vehicle and microinjection of drugs into the NTS (D). R: recording 3swallows evoked by the SLN stimulation, WIN: WIN 55,212-2, CB1 ant: AM 251, CB2 ant.: AM 630, i.v.: intravenous infusion.

**Figure 2:**
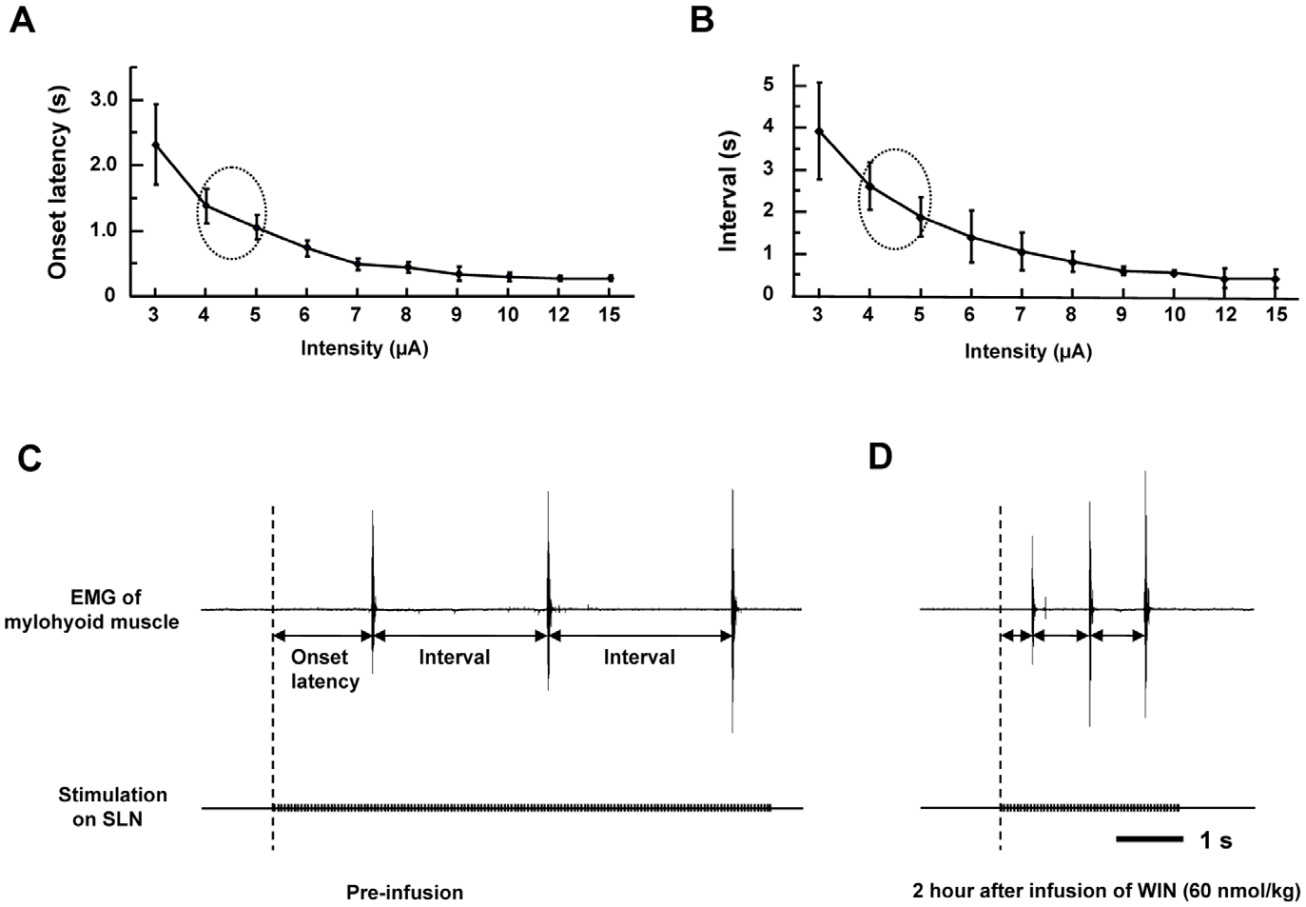
Example of facilitation of the electrically-stimulated swallowing reflex by the cannabinoid agonist, WIN 55,212-2. The onset latency to the first swallow (A) and intervals between swallows (B) induced by different intensities of electrical stimulation of the SLN of naïve rats (n = 5). The ellipses indicate the characteristics of the swallowing reflex by the electrical stimulation at 4–5 µA. The typical example that the swallowing reflex by the SLN stimulation before (C), and two hours after (D), infusion of WIN 55,212-2. Note that WIN 55,212-2 decreases the onset latency to the first swallow and the intervals between swallows.

First, the SLN in naïve rats was stimulated by repetitive electrical stimulation with various stimulus intensity (intensity: 3–15 µ*A*, frequency: 20 Hz, duration: 1.0 ms) in order to investigate relationships between the intensity of electrical stimulation and the properties of the swallowing reflex (Fig. 1A). After measuring the relationship of the stimulus intensity to onset latency and intervals between the swallowing reflexes, the parameter of the electrical stimulation (intensity: 4–5 µA, frequency: 20 Hz, duration: 1.0 ms) was adjusted to keep the onset latency for the first swallow between 1 and 1.5 s for the experiment (Fig. 2A). The swallowing reflexes were evoked before and after the drug infusion and electrical stimulation was discontinued after three consecutive swallows were evoked for each time point (Fig. 2C and 2D). The onset latency of the first swallow and the time interval between swallow was analyzed. The onset latency of the first swallow was defined as the time required for evoking the first swallow after the onset of electrical stimulation. The time intervals between the first and second swallows and second and third swallows were averaged and the averaged time was considered the time interval between swallows. The onset latency of the first swallow and the time interval between swallows were averaged from three electrical stimulations.

The onset latency of the first swallow and the time interval between swallows calculated were measured before and after intravenous infusion of the drugs or vehicle (see Fig. 1B, 1C and 1D).

### Drugs and Chemicals

To investigate the dose dependent effect of the cannabinoid, the cannabinoid receptor agonist, WIN 55,212-2 (Sigma-Aldrich, USA), was used at various concentrations (60, 30 and 15 nmol/kg). The CB1 receptor antagonist, AM 251 (120 nmol/kg as pretreatment, 120, 250 and 500 nmol/kg when used alone, in addition, 12 pmol in 100 nl to use microinjection), and the CB2 receptor antagonist, AM 630 (500 nmol/kg), were both acquired Wako Pure Chemical Industries, Ltd. (Osaka, Japan). The drugs were made up in vehicle comprising ethanol (5%), Tween-80 (10%), and normal saline (85%). The drugs were infused at a volume of 1 ml/kg.

### Microinjection into the NTS

To investigate the role of CB1 receptors in the NTS, the drugs were microinjected into the NTS. For this experiment, the animals were placed in the prone position and a craniotomy was performed to expose the NTS. However, in the prone position, it was difficult to keep an electrode fixed in order to stimulate the SLN. Therefore, we initially microinjected the CB1 receptor antagonist into the NTS in the supine position, and then intravenously administered the CB1 receptor agonist after setting up of the electrode for SLN stimulation in the prone position.

Rats were fixed in the prone position using the stereotaxic frame. The CB1 receptor antagonist, AM 251 (12 pmol, 100 nl) was microinjected stereotaxically in the central pattern generator of swallowing, which is located in the region of the STN extending between 0.5 and 0.7 mm rostral to the caudal edge of the area postrema (taken as 0), 0.6–0.8 mm laterally, and 0.6–0.8 mm in depth, according to a previous study [28]. The rats were then placed in the supine position and the CB1 receptor agonist, WIN 55,212-2 (30 nmol/kg), was intravenously administered 15 min after the microinjection of AM 251 into the NTS. By this time, we had also placed stimulating electrodes on the SLN. The SLN was stimulated electrically to elicit the swallowing reflex (intensity: 4–5 µA, frequency: 20 Hz, duration: 1.0 ms) (Fig. 1). The effect of this stimulus on the swallowing reflex was intended to elicit the onset latency of 1–1.5 s in naïve rats (Fig. 2A). As the contrast experiment, the vehicle (100 nl) was microinjected into the NTS before the intravenous infusion of WIN 55,212-2 (30 nmol/kg) (Fig. 1).

The onset latency and intervals between evoked swallows were analyzed at different time points for 3 h.

### Immunohistochemistry

For double immunofluorescence histochemistry, rats were deeply anesthetized with sodium pentobarbital and perfused with 200 ml of phosphate-buffered saline (PBS) followed by 500 ml of 4% paraformaldehyde. The hindbrain was removed and post-fixed in the same fixative for 2 days and was then transferred to 20% sucrose (w/v) in PBS for several days to ensure effective cryoprotection. Sections (30 µm) were cut on a freezing microtome and were collected in PBS. Free-floating tissue sections were rinsed in PBS and blocked with 10% normal goat serum (NGS) in 0.3% Triton X-100 for 1 h to prevent non-specific staining. The sections were then incubated with rabbit polyclonal anti-CB1 receptor antibody (1∶200; Abcam, USA) and either mouse monoclonal anti-GAD67 antibody (1∶600; Sigma, USA) or mouse anti-glutamate antibody (1∶600, Millipore, USA) in 3% NGS and 0.3% Triton X-100 at 4°C for 48 h. After washing in PBS, the sections were incubated with Alexa Flour 488 goat anti-rabbit IgG (1∶1000, Invitrogen, USA) and Alexa Flour 568 goat anti-mouse IgG (1∶200, Invitrogen, USA) for 2 h. Sections were subsequently washed in PBS, mounted on slides and coverslipped with VECTASHIELD Mounting Medium (Vector Lab., USA). Images of immunofluoresence staining were photographed with a camera attached to a fluorescent microscope (Biozero 8000, Keyence Corp., Japan).

Co-localization of CB1 receptor immunoreactivity in glutamic acid decarboxylase 67 (GAD67)-immunoreactive (ir) or glutamate-ir cells was assessed throughout the NTS. To understand the rostrocaudal distribution of CB1 receptors in the gamma-aminobutyric acid (GABA)ergic and glutamatergic neurons, sections were compared with the Chemoarcitectonic Atlas of the Rat Brainstem [29] and divided into four rostrocaudal levels: most rostral, rostral, intermediate, and caudal. Similar divisions have been made in previous studies [30], [31]. The most rostral level was approximately between bregma –12.36 mm and bregma –12.84 mm and the rostral level was approximately between bregma –12.84 mm and bregma –13.56 mm. The intermediate level was at the level of the area postrema, approximately between bregma–13.56 mm and bregma –14.16 mm. The caudal level was defined as the level caudal to the area postrema, between bregma –14.16 mm and bregma –14.65 mm. To assess the co-localization CB1 receptors with GAD67 or glutamate, double labeled cells were identified and analyzed using a BZ-8000 system (Keyence, Osaka, Japan). The ir-cells showing staining two times more intense than the average background (yellow labeled cells) were considered positive for co-localization. Three sections were randomly chosen from each level and the number of GAD67-ir and glutamate-ir cells in which CB1 receptor immunoreactivity co-localized were counted throughout the rostrocaudal axis or the lateral and medial portion of the NTS, using Image J software (NIH, USA). The average of the 3 sections was used for statistical analysis.

### Statistical Analysis

The pre-infusion data was compared with post-infusion data using ANOVA followed by Dunnett’s post-hoc test. ANOVA followed by Student-Newman-Keuls post-hoc test were performed when comparing the data for the effects of the different doses on WIN 55,212-2. The immunohistochemical data was compared using the Student’s *t*-test. Data are expressed as mean ± SD.

## Results

### Effect of the Cannabinoid Receptor Agonist, WIN 55,212-2, on the Swallowing Reflex

To determine the electrical stimulus intensity, we analyzed the relationship between stimulus intensity with the onset latency and interval of the swallowing reflex in naïve rats (Fig. 2A and 2B). Both onset latency and interval decreased with increased stimulus intensity. It was observed that onset latency decreased at around 1 to 1.5 s at 4–5 µA, (the ellipses in Fig. 2A and 2B). Although the onset latency of the swallowing decreased with increased stimulus intensity, changes in the swallowing latency due to stimulus intensity 7–15 µA were not significantly different. Therefore, we decided to use the stimulus intensity 4 to 5 µA in the present study.

The swallowing reflex was evoked before and after infusion of the drugs in anesthetized rats. Typical examples of the facilitation of the swallowing reflex after the intravenous infusion of WIN 55,212-2 (60 nmol/kg) is shown in Fig. 2C and 2D. Before the intravenous infusion of WIN 55,212-2, the onset latency and interval were 1.46 s and 2.62 s, respectively (Fig. 2C). Two hours after the intravenous infusion of WIN 55,212-2 (60 nmol/kg), the onset latency and interval decreased to 0.45 s and 0.85 s, respectively (Fig. 2D).

**Figure 3:**
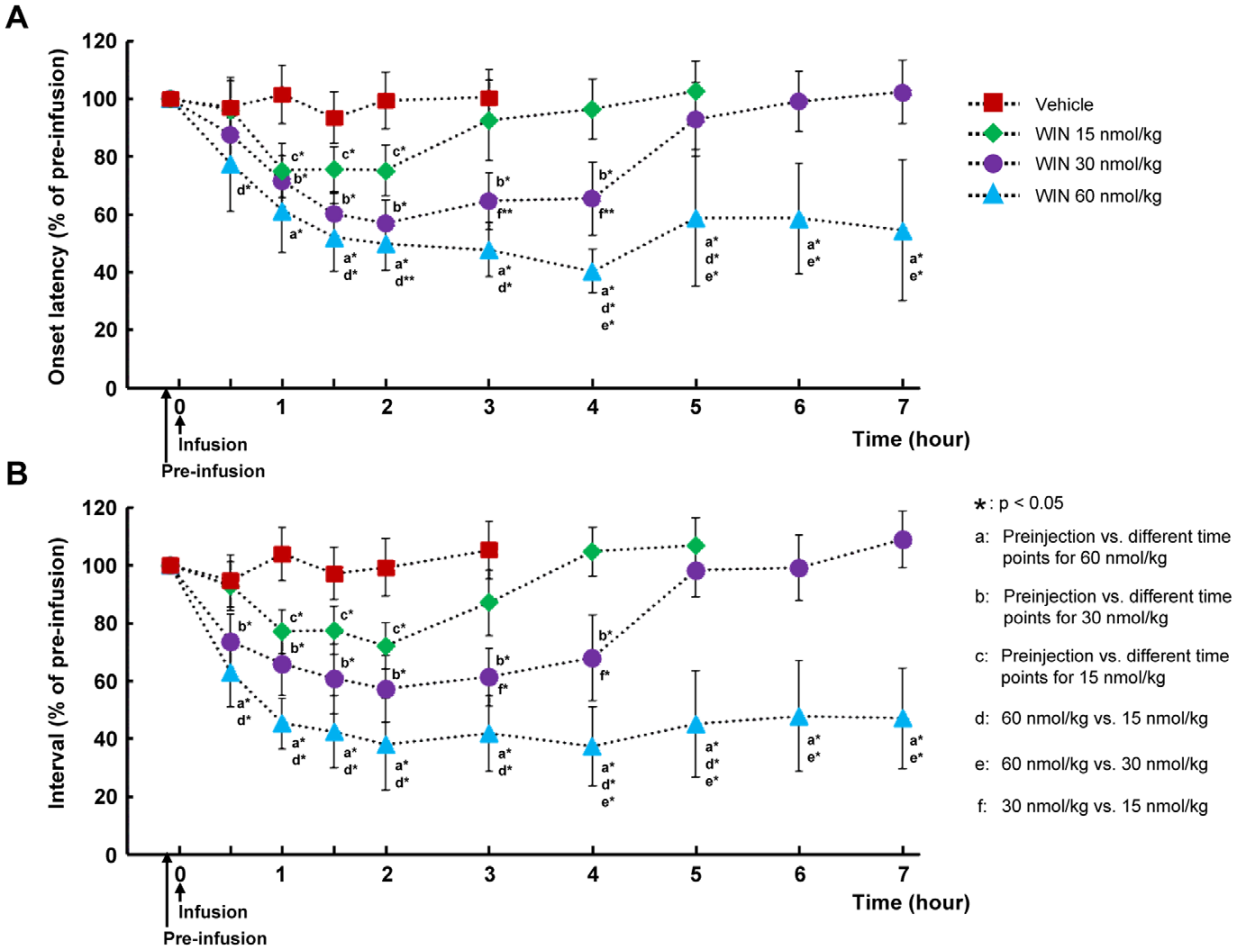
Effect of different doses of cannabinoid receptor agonist on the onset latency to the first swallow and intervals between swallows. Graphs demonstrating the effect of WIN 55,212-2 on the onset latency to the first (A) and intervals between electrically stimulated swallows (B). Dose-dependent facilitation of the electrically stimulated swallows by WIN 55,212-2 was observed. Data are presented as percentage of pre-infusion data and n = 5 for each group.

Figure 3 shows the effect of different doses of the intravenous infusion of WIN 55,212-2 on the onset latency of the first swallow and the intervals between swallows. The effect of WIN 55,212-2 on the swallowing reflex was specific (the vehicle alone did not alter either the onset latency or the interval) and dose-dependent (Fig. 3). The facilitatory effects of WIN 55,212-2 on the latency and the interval of the swallowing reflex were observed. At dose of 60 nmol/kg, the onset latency (Fig. 3A) and interval (Fig. 3B) significantly decreased 30 min after the intravenous infusion of WIN 55,212-2. The strong facilitatory effect of WIN 55,212-2 was observed 1 h after infusion, and the effect persisted at least 7 h after the infusion of WIN 55,212-2. After treatment with WIN 55,212-2 at a dose of 30 nmol/kg, the onset latency significantly decreased from 1 h to 4 h after infusion (Fig. 3A). In addition, the interval was significantly decreased from 30 min to 4 h after the infusion, but returned to pre-infusion levels after 4 h of infusion (Fig. 3B). At the 15 nmol/kg dose, the onset latency and interval were significantly decreased only from 1 h to 2 h after infusion of WIN 55,212-2. Significant differences were also observed in the onset latency and interval at various time points between the doses of 60 nmol/kg and 30 nmol/kg, and between 30 nmol/kg and 15 nmol/kg (Fig. 3).

### Effect of Pretreatment with CB1 or CB2 Antagonist on the Swallowing Reflex

**Figure 4:**
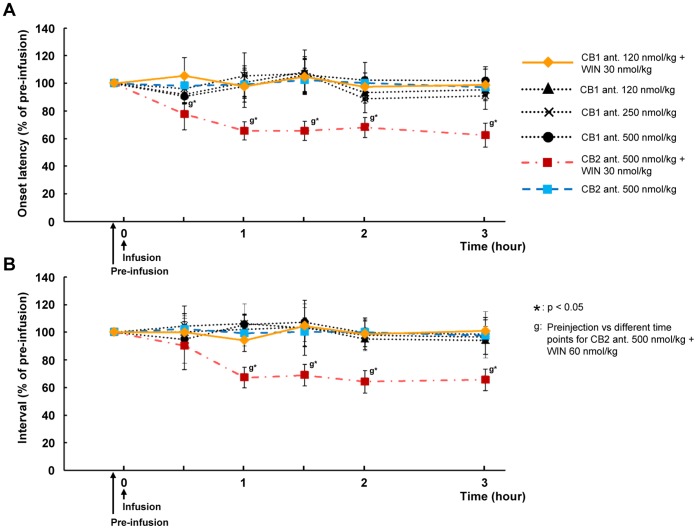
Effect of different doses of cannabinoid receptor antagonists on the onset latency to the first swallow and intervals between swallows. Graphs demonstrating the effect of combination of WIN 55,212-2 and cannabinoid (CB1 or CB2) receptor antagonists, and different doses of cannabinoid receptor antagonists on the onset latency to the first (A) and intervals between (B) electrically stimulated swallows. Pretreatment with a CB1 receptor antagonist blocked the effect of WIN 55,212-2, whereas pretreatment with a CB2 receptor antagonist did not. The CB1 antagonist alone and the vehicle had no significant effect on the swallowing reflex. Data are presented as percentage of pre-infusion data and n = 5 for each group. CB1 ant: CB1 receptor antagonist (AM 251), CB2 ant.: CB2 receptor antagonist (AM 630).

To investigate whether the facilitatory effect of WIN 55,212-2 is mediated through the CB1 or CB2 receptors, CB1 and CB2 receptor antagonist was administrated prior to WIN 55,212-2. When the CB1 receptor antagonist (AM 251: 120 nmol/kg) was intravenously administered 15 min prior to infusion of WIN 55,212-2 (30 nmol/kg), the facilitatory effect of WIN 55,212-2 on the swallowing reflex was attenuated and no significant modulation of the swallowing reflex was observed over a 3 h period (Fig. 4). In contrast, when the CB2 receptor antagonist (AM 630: 500 nmol/kg) was intravenously administered 15 min prior to infusion of WIN 55,212-2 (30 nmol/kg), the WIN 55,212-2-mediated facilitation of the swallowing reflex was preserved (Fig. 4).

In addition, data was rerecorded for 3 h following intravenous administration of the CB1 receptor antagonist (AM 251: 120, 250 and 500 nmol/kg) or CB2 (AM 630: 500 nmol/kg) alone. There was no significant influence on the onset latency or interval of the swallowing reflex as compared with those observed before treatment (Fig. 4).

### Microinjection of the CB1 Receptor Antagonist into the NTS Prior to Intravenous Infusion of WIN 55,212-2

**Figure 5:**
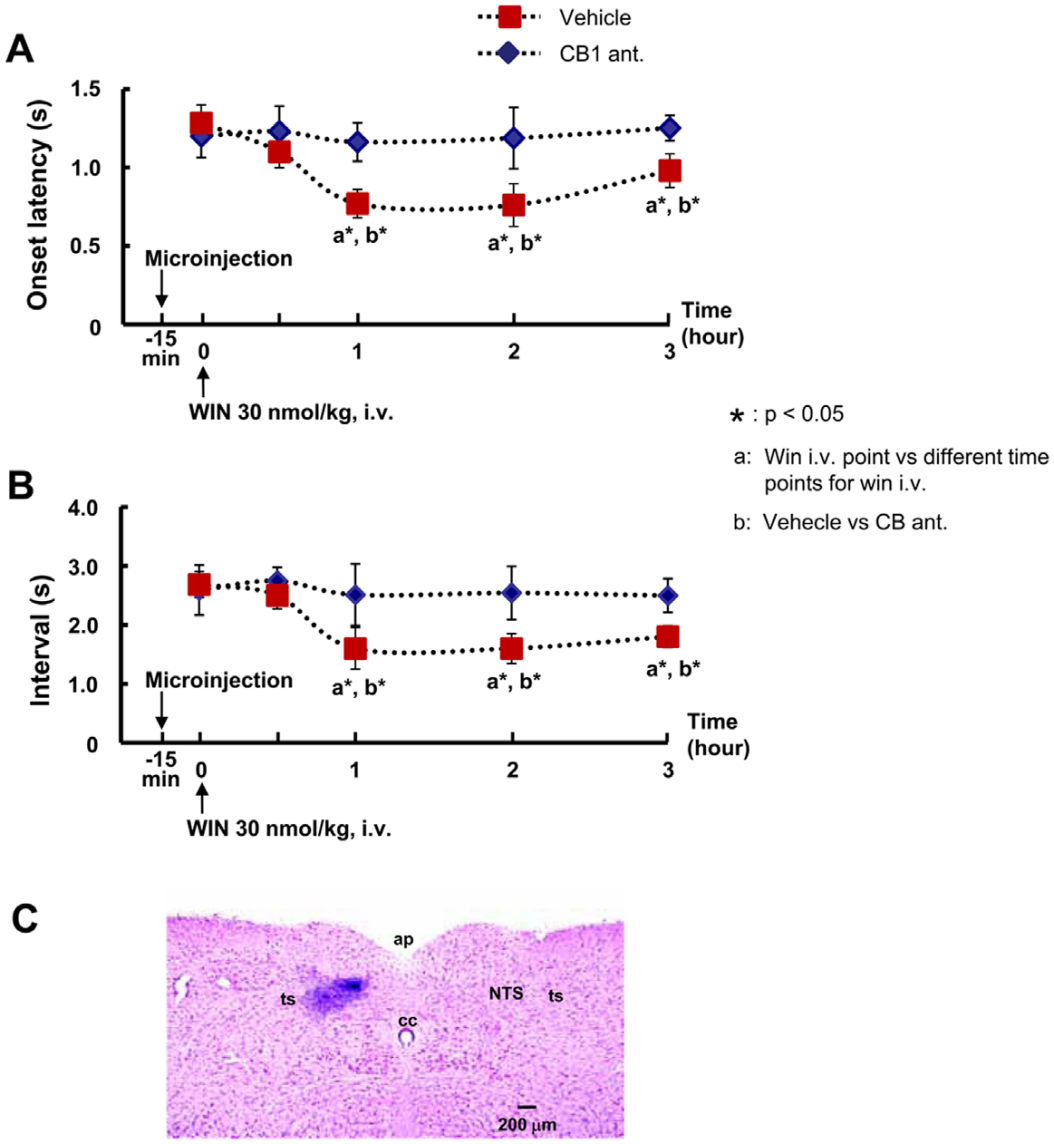
Effect of microinjection of CB1 receptor antagonist (AM 251) into the NTS prior to intravenous infusion of WIN 55,212-2. Microinjection of AM251 blocked the enhancement of the onset latency to the first swallow (A) or the intervals between swallows (B) in the swallowing reflex by WIN 55,212-2 intravenous infusion. Note that the facilitatory effect of WIN 55,212-2 on electrically stimulated swallows was persisted after the microinjection of vehicle in NTS. The photomicrograph of the microinjection site of drugs in the NTS (C). WIN: WIN 55,212-2, CB1 ant.: CB1 receptor antagonist (AM 251).

In order to investigate the possible role of the CB1 receptors in the NTS in the facilitation of swallowing after intravenous infusion of WIN 55,212-2, CB1 antagonist (AM 251: 12 pmol, 100 nl) or vehicle (100 nl) was microinjected into the NTS 15 min before WIN 55,212-2 administration. Figure 5A and 5B shows the onset latency of the first, and the intervals between swallows. As mentioned in the intravenous administration of CB1 antagonist, the microinjection of CB1 antagonist into the NTS also completely blocked the effect of WIN 55,212-2, and there was no significant facilitation in latency or interval of the swallowing reflex. However, the microinjection of vehicle (100 nl) of AM 251 into the NTS did not block the effect of WIN 55,212-2, and there was a significant facilitatory effect of WIN 55,212-2 on the latency and interval of the swallowing. The location of the microinjection within the NTS was confirmed by adding 4% pontamine sky blue with AM 251 or the vehicle in all rats, and the histology of the brainstem was conducted for localization of the injection site. In all rats, the injection site was localized within the NTS (Fig. 5C).

### Co-localization of CB1 Receptor Immunoreactivity with GAD67- and Glutamate-ir Cells in the NTS

**Figure 6:**
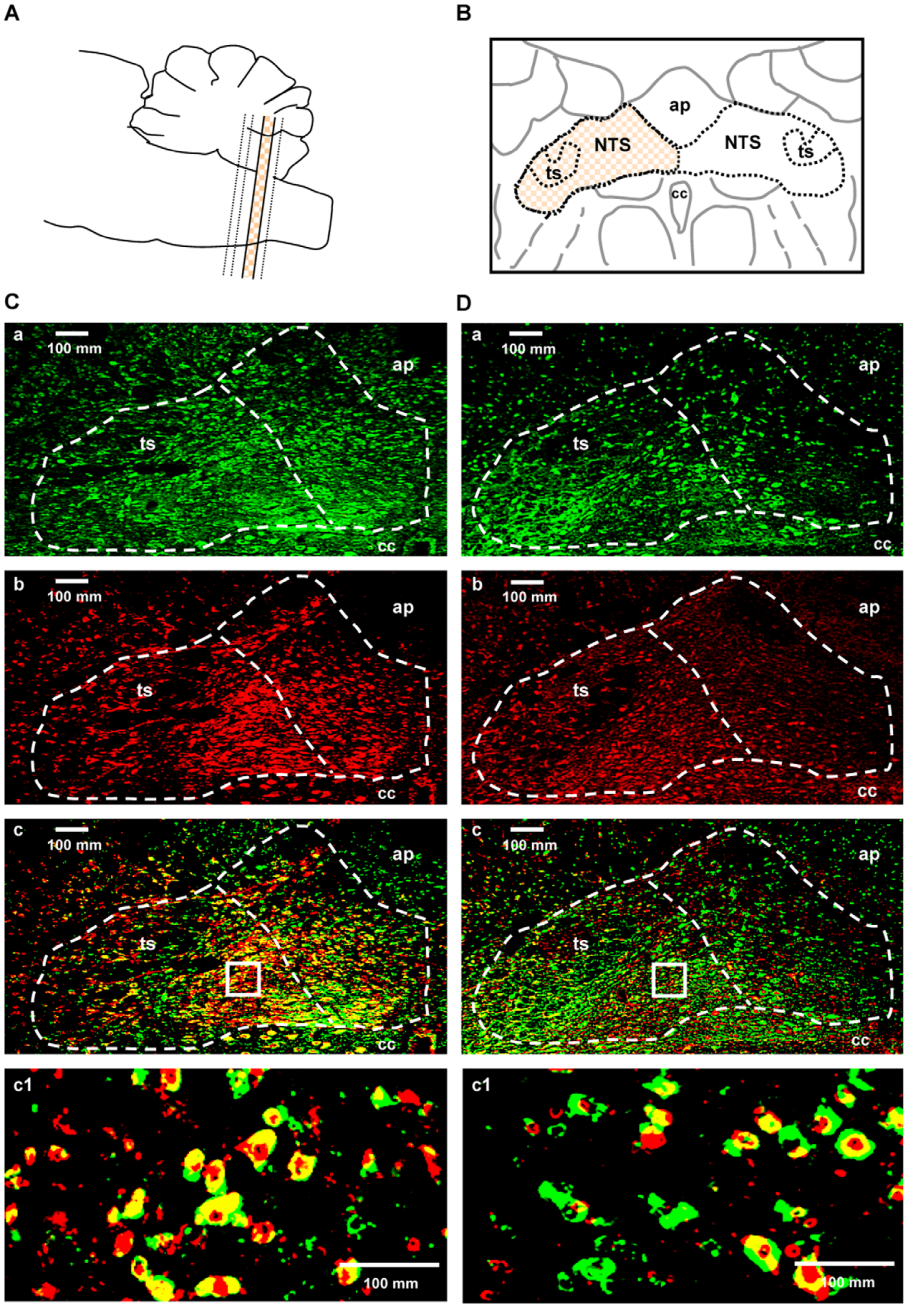
Photomicrographs of immunoreactivity for CB1, GAD67 and glutamate in the NTS. The illustrations show the intermediate area (the mosaic and beige colored part) divided into four rostrocaudal levels in the NTS (A and B). Photomicrographs of immunoreactivity for CB1 receptors (Ca and Da), GAD67 (Cb), glutamate (Db), CB1 receptors + GAD67 (Cc) and CB1 receptors + glutamate (Dc) in the NTS. The left and right area surrounded by the broken line in Ca, Cb, Cc and Da, Db, Dc indicates the lateral and medial portion of the NTS, respectively. The small square boxes in Cc and Dc shows the area of the high magnified images that in Cc1 and Dc1. High magnified images demonstrating co-localization of CB1 receptors + GAD67 and CB1 receptors + glutamate are shown in Cc1 and Dc1, respectively. ap: area postrema, ts: tractus solitaries, cc: central canal.

**Figure 7:**
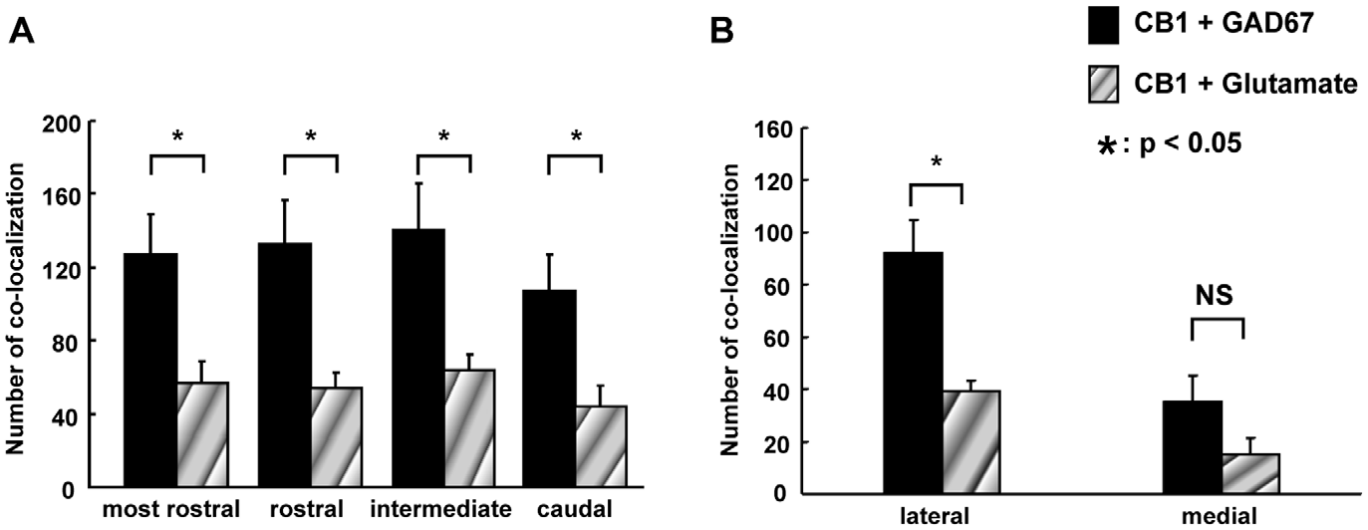
Co-localization of CB1 receptors + GAD67 and CB1 receptors + glutamate immunoreactivity. Quantification of the co-localization of CB1 receptor immunoreactivity with GAD67 and glutamate immunoreactivity in the rostrocaudal direction of the NTS (A). CB1 receptor immunoreactivity was observed to be co-localized with significantly more GAD67-ir cells than glutamate-ir cells throughout the rostrocaudal axis. The number of cells in which CB1 receptor immunoreactivity co-localized with both GAD67 and glutamate immunoreactivity was greater in the lateral portion than the medial portion of the NTS (B).

Figure 6A illustrates the rat brainstem sections divided into four rostrocaudal levels (the most rostral, rostral, intermediate and caudal). The mosaic patterned areas in Fig. 6A and 6B indicate the intermediate levels, respectively. A double immunofluorescence histochemical study was conducted to visualize CB1 receptor immunoreactivity, GAD67- and glutamate-ir cells in the NTS. Typical pictures of the intermediate level show CB1 receptor immunoreactivity (Fig. 6Ca and 6Da), GAD67-ir cells (Fig. 6Cb) and glutamate-ir cells (Fig. 6Db) in the NTS. CB1 receptor immunoreactivity was found to be co-localized with GAD67- ir cells (Fig. 6Cc and 6Cc1) and glutamate-ir cells (Fig. 6Dc and 6Dc1) in the NTS. We analyzed the number of co-localization of CB1 receptor immunoreactivity with GAD67- and gulutamate-ir cells throughout the rostrocaudal axis or the lateral and medial portion of the NTS. Co-localization occurred significantly more in GAD67-ir than glutamate-ir cells throughout the rostrocaudal axis of the NTS (Fig. 7A). Moreover, co-localization of CB1 with GAD67- ir cells was found more prevalent than that of CB1 with gulutamate-ir cells in the lateral portion of the NTS (Fig. 7B).

## Discussion

The present study shows that the cannabinoid receptor agonist, WIN 55,212-2, enhances the swallowing reflex elicited by electrical stimulation of the SLN. The duration of the effect of WIN 55,212-2 on the electrically stimulated swallowing reflex was dose-dependent. The onset latency of the first swallow and the interval between swallows was significantly shorter in rats which received higher doses of WIN 55,212-2 than those in the lower dose group. Immunohistochemical studies have revealed that CB1 receptors are located in the NTS where they co-localize more with GABAergic neurons than with glutamatergic neurons. In addition, in the lateral part of the NTS a significantly greater number of the GABAergic neurons with CB1 receptors were found than glutamatergic neurons with CB1 receptors.

Electrical stimulation of the SLN reliably elicits the swallowing reflex [23], [24], [25], [26], [27]. Afferents fibers of the SLN project to the NTS [22], [32]. Several reports indicate that NTS neurons play a leading role in the generation of swallowing [22]. Sequential and rhythmic firing of neurons in the NTS have been shown to parallel the motor pattern of swallowing [33], [34], [35]. Pre-swallowing neuronal activity is also located mostly within the NTS [22]. Moreover, systematic studies of the brainstem indicate that central areas responsible for swallowing are situated in the region of the NTS [33], [36]. Modulation of the swallowing reflex using fine microinjections of drugs into the NTS also provides firm evidence that the central pattern generator of swallowing is located in the NTS [37], [38], [39], [40]. In the present study we also observation that NTS may play a major role in the modulation of the swallowing reflex after the administration of cannabinoid.

It has been postulated that the lateral portion of the NTS comprising the intermediate, central, interstitial, ventral and ventrolateral subdivisions of the nucleus may be involved in the triggering and programming of swallowing [22], [33], [41], [42]. However, complex neuronal connections exist among the various subnuclei of the NTS [35], [41], [43], suggesting the involvement and integration of neurons of different subnuclei. Several neurotransmitter systems have been reported to play a role in the swallowing mechanism [22], [44], [45]. Among them, glutamatergic and GABAergic neurons play a major role. In consideration of the present study, it is possible that lateral portion of the NTS is involved in the facilitation of the swallowing reflex mechanism.

Some recent studies have reported that endogenous cannabinoid (2-arachidonoylglycerol: 2-AG) is synthesized in, and released from post-synaptic neurons. The binding of 2-AG to CB1 receptors on pre-synaptic neurons causes suppression of neurotransmitter release. This novel neuroregulatory system is called “Retrograde suppression of synaptic transmission” [46], [47].

In the present study, we observed that the non-selective cannabinoid receptor agonist WIN 55,212-2 facilitated the electrically stimulated swallowing reflex in a dose-dependent manner. When the CB2 receptor antagonist, AM 630, was infused prior to WIN 55,212-2, the facilitation of the electrically stimulated swallowing reflex was unaffected. In contrast, pretreatment with the highly selective CB1 receptor antagonist AM 251 effectively blocked the effect of WIN 55,212-2. In addition, the dose-response study of AM 251 (120, 250 and 500 nmol/kg) had no significant influence on the onset latency and interval time of the swallowing reflex induced by electrical stimulation as compared with those observed before treatment. Together, these results strongly suggest that the action of WIN 55,212-2 is mediated by CB1 receptors. However, infusion of the CB1 receptor antagonist alone did not have any effect on swallowing. CB1 receptor antagonists administered alone have previously been found to produce no effect in many studies where antinociceptive or antiemetic effects of cannabinoids were investigated [14], [15], [16], [48], [49], [50], [51], [52], [53]. From the above observations, it can be assumed that WIN 55,212-2 binds to pre-synaptic CB1 receptors, and results in the suppression of neurotransmitter release. Intravenously infused drugs spread to various areas of the brain and periphery. The central pattern generator of swallowing located in the NTS receives input from peripheral afferents and various supramedullary areas [22]. The above observation is supported by the present findings that the intravenous infusion of WIN 55,212-2 modulates the swallowing reflex. However, the modulatory effect of WIN 55,212-2 was blocked by microinjection of CB1 antagonist in the NTS. The present study shows that the effect of WIN 55,212-2 on the swallowing reflex is probably expressed in the central pattern generator of swallowing in the NTS.

Previous studies have reported that CB1 receptors were present in the NTS [8], [12], [14]. Regarding this, Partosoedarso and collaborators (2003) showed that vagotomy or nodose ganglionectomy did not alter the expression of the CB1 receptor in the DVC of ferret, including in the NTS. Consistent with the above findings, our immunohistochemical study also showed that CB1 receptors are abundant in the NTS.

Regarding neurotransmitter involvement, we have found higher co-localization of CB1 receptors with GABAergic neurons than with glutamatergic neurons, suggesting that cannabinoids may have a greater inhibitory effect on GABA release than on glutamate release in the NTS. Therefore, the excitatory effects are higher than the inhibitory effects which may result in the facilitation of the swallowing reflex. Moreover, expression of CB1 receptors was observed in more GABAergic and glutamatergic neurons in the lateral portion of the NTS, the area that been particularly implicated in the process of swallowing. The present study suggests that the mechanism by which WIN 55,212-2 enhances the electrically stimulated swallowing reflex may include the attenuation of tonic GABAergic input in the lateral portion of the NTS. Another study using brainstem slices showed that both inhibitory and excitatory synaptic inputs in the dorsal motor nucleus of the vagus originating from the NTS were attenuated by WIN 55,212-2 [11]. This study suggested that activation of CB1 receptors attenuates the release of neurotransmitters from the terminals of NTS neurons that regulate parasympathetic motor output [11].

Emetic stimulation has been found to inhibit the swallowing reflex in decerebrate rats [54]. As cannabinoids inhibit emesis [14], [15], [16], [17], it can be assumed that cannabinoids facilitate the swallowing reflex. In addition, leptin (a hormone secreted by adipose tissue) has been found to inhibit the swallowing reflex by inhibiting the activity of the premotoneurons responsible for swallowing [28]. Leptin is a satiety signal that acts on the hypothalamus to reduce food intake [6], [55]. An inverse functional relationship between cannabinoids and leptin has been found where leptin administration suppresses the expression of endogenous cannabinoids in the hypothalamus of normal rats. While genetically obese, chronically hyperphagic rats showed elevated levels of endogenous cannabinoids [56]. Considered with our data, these studies demonstrate that cannabinoids can both regulate food intake and facilitate swallowing.

Our finding that cannabinoids can modulate the swallowing reflex has physiological significance. It suggests that cannabinoids regulate swallowing via the central pattern generator of swallowing in the NTS, using the retrograde suppression of synaptic transmission. In the biological condition, it may be possible that endogenous cannabinoid (2-AG) is involved in the fine-tuning of motor output from the central pattern generator of swallowing by modulating synaptic neurotransmitter release. This phenomenon in the central pattern generator of swallowing may controls the daily regulation of the swallowing reflex.

In conclusion, the present study demonstrates that cannabinoids facilitate the electrically stimulated swallowing reflex. CB1 receptors located in the central pattern generator of swallowing in the NTS are likely to be involved in the facilitation of the mechanism. In addition, the findings of the present study also raise the possibility of the therapeutic use of cannabinoid ligands for treating dysphagia (difficulty swallowing), although further studies are required in this regard.
